# AMPK-mediated autophagy is involved in the protective effect of canagliflozin in the vitamin D3 plus nicotine calcification model in rats

**DOI:** 10.1007/s00210-023-02627-x

**Published:** 2023-07-31

**Authors:** Wafaa A. Hewedy, Shaymaa A. Abdulmalek, Doaa A. Ghareeb, Esraa S. Habiba

**Affiliations:** 1 Clinical Pharmacology Department, Faculty of Medicine, Alexandria University, Alexandria, Egypt; 2 Al-Moassat Medical Campus, Elhadara, Clinical Pharmacology Department, Faculty of Medicine, Alexandria University, 21561 Alexandria, Egypt; 3 Bio-Screening and Preclinical Trial Lab, Biochemistry Department, Faculty of Science, Alexandria University, Alexandria, Egypt

**Keywords:** AMPK, Autophagy, Canagliflozin, Nicotine, Vascular calcification, VitaminD3

## Abstract

Vascular calcification (VC) is a major risk factor for cardiovascular events. A mutual interplay between inflammation, oxidative stress, apoptosis, and autophagy is implicated in its development. Herein, we aimed to evaluate the potential protective effects of canagliflozin in a vitamin D3 plus nicotine (VDN) model of VC, and to explore potential mechanisms. VC was induced by VDN in adult male Wistar rats on day one. Then, rats were randomly assigned into three groups to receive canagliflozin (10 mg or 20 mg/kg/day) or its vehicle for 4 weeks. Age-matched normal rats served as a control group. After euthanization, aorta and kidneys were harvested for biochemical and histopathological evaluation of calcification. Aortic markers of oxidative stress, alkaline phosphatase (ALP) activity, runt-related transcription factor (Runx2) and bone morphogenic protein-2 (BMP-2) levels were determined. Additionally, the protein expression of autophagic markers, LC3 and p62, and adenosine monophosphate activated protein kinase (AMPK) were also assessed in aortic homogenates. Canagliflozin dose-dependently improved renal function, enhanced the antioxidant capacity of aortic tissues and reduced calcium deposition in rat aortas and kidneys. Both doses of canagliflozin attenuated ALP and osteogenic markers while augmented the expression of autophagic markers and AMPK. Histopathological examination of aortas and kidneys by H&E and Von Kossa stain further support the beneficial effect of canagliflozin. Canagliflozin could alleviate VDN-induced vascular calcification, in a dose dependent manner, via its antioxidant effect and modulation of autophagy. Further studies are needed to verify whether this effect is a member or a class effect.

**Supplementary information:**

The online version contains supplementary material available at 10.1007/s00210-023-02627-x.

## Introduction

Vascular calcification (VC) refers to the deposition of mineral salts, mainly hydroxyapatite crystals, in the vessel wall. It is the most frequent site of ectopic soft tissue calcification that has been recognized as one of the characteristic morphological signs of vascular aging (Lee et al. 2020). However, VC is also known to be accelerated in diabetes mellitus (DM), dyslipidemia, and end-stage renal disease (Ghosh et al. 2020). Once developed, VC accounts for devastating functional changes in the vascular system including gradual loss of vascular compliance necessary for adequate tissue perfusion, high systolic pressure, and ventricular overload that ultimately leads to increased risk of adverse cardiovascular events (Johnson et al. 2006; Lee et al. 2020).

Two distinct patterns of VC have been described, intimal calcification and medial calcification. The former is common with atherosclerotic lesions, while the latter is associated with aging and chronic morbid conditions. Medial calcification occurs within elastic lamellae of affected arteries where the vascular smooth muscle cells (VSMCs) undergo a phenotypic transition from contractile cells into osteogenic cells that express key osteogenic markers and become capable of hydroxyapatite formation (Johnson et al. 2006) (Wu et al. 2013).

The pathogenesis of VC is incompletely understood. Two signaling cascades have been described as the master regulator for osteoblastogenesis, bone morphogenic (BMP)/SMAD signaling pathway and Wnt/β-catenin pathway. They regulate the expression of osteoblastic transcription factors, runt-related transcription factor 2 (Runx2) and osterix. In turn, RUNX2 and osterix orchestrate the expression of several bone-related proteins including sclerostin, osteocalcin, and osteopontin which trigger the osteogenic transition of VSMCs (Johnson et al. 2006). Ultimately, VSMCs transdifferentiation into osteoblastic phenotype is evidenced by the expression of alkaline phosphatase (ALP), a marker of functional osteoblasts, that catalyzes dephosphorylation of inorganic pyrophosphate (PPi) with subsequent deposition of hydroxyapatite crystals in the calcific vasculature (Vattikuti and Towler 2004) (Liu et al. 2018).

Several mechanisms have been suggested to explain the development of VC. Oxidative stress and overproduction of proinflammatory cytokines are initially introduced as main mechanisms (Shen et al. 2021) (Steven et al. 2019). They are known to be involved in the pathogenesis of chronic conditions associated with VC. Further, they can activate the upstream regulators of osteoblastogenesis, mainly BMPs, to trigger transdifferentiation of VSMCs to osteogenic phenotype (Yamada et al. 2016) (Watanabe et al. 2020).

Defective autophagy has emerged as another proposed mechanism for VC development and progress. Autophagy is a dynamic process that recycles intracellular components, via a lysosome-mediated mechanism, to get rid of fractious or dysfunctional proteins, and provide cells with an alternative source of nutrients and energy under stressful conditions. Autophagic machinery is maintained at a low basal level under normal conditions. Alternatively, under stressful conditions, autophagy is upregulated to safeguard cell survival. Impaired autophagy, on the other hand, is detrimental. It has been demonstrated in various pathological conditions including cancer, cardiovascular, metabolic, and neurodegenerative diseases (Levy et al. 2017; Moors et al. 2017; Packer 2020). Hence, targeting autophagy is investigated as a potential treatment strategy of several pathological conditions.

Accumulating data points to the crucial role of autophagy in the regulation of Ca^2+^ flux and homeostasis in VSMCs, and hence impacts vascular reactivity (Grootaert et al. 2015) (Phadwal et al. 2020). Furthermore, induction of autophagy was demonstrated to inhibit the differentiation of VSMCs into osteogenic lineage (Frauscher et al. 2018) (Phadwal et al. 2020). Thus, the realization of the critical role of autophagy in VC paves the way for several studies to investigate the utility of pharmacological modulation of autophagy on progression VC (Phadwal et al. 2020). On the other hand, the underlying molecular mechanisms that regulate autophagy are complex. The low-energy sensors, sirtuin-1 (SIRT1) and adenosine monophosphate-activated protein kinase (AMPK) are known to be involved. They can regulate the transcription of certain genes in an attempt to preserve cell survival over cell growth (Lee et al. 2020) (Pietrocola and Pedro 2021).

Inhibitors of sodium-glucose cotransporter-2 (SGLT2) are the most recent approved antidiabetic drugs. In addition to their well-documented metabolic effects, SGLT2 inhibitors show beneficial effects on cardiovascular tissues (Takata and Isomoto 2021).They were initially thought to exert favorable effect on the vasculature and mitigate arterial stiffness owing to their hypoglycemic effect (Xiao et al. 2021). However, they have also been demonstrated to decrease the production of proinflammatory cytokines, and reactive oxygen species (ROS) while increasing nitric oxide (NO) bioavailability in endothelial cells. Hence, they are expected to improve vascular reactivity and reprieve vascular aging (Soares et al. 2022).

The role of SGLT inhibitors needs to be further investigated beyond their hypoglycemic effect. Accordingly, in the current study we sought to characterize the possible protective effect of canagliflozin, an SGLT inhibitor, in VDN-induced calcification. Previous studies demonstrated that a supraphysiological dose of vitamin D plus nicotine (VDN) is associated with ectopic calcification of vessel walls (Niederhoffer et al. 1997). This VC model shows pathological features that mirror calcification associated with aging, diabetes, and chronic renal diseases. Given that canagliflozin is the only SGLT2 inhibitor that activates AMPK, we also aimed to determine whether AMP signaling-dependent autophagy is involved in such effect.

## Materials and Methods

### Ethics statement

All animal care and experimental procedures were approved by the Ethical Committee of the Faculty of Medicine, Alexandria University, Alexandria, Egypt (IRB NO: 00012098-FWA NO: 00018699) and complied with the Guide for the Care and Use of Laboratory Animals published by the US National Institutes of Health (NIH Publication no. 85–23, revised 1996).

### Animals

Thirty-two adult male Wistar rats (100–130 g) were obtained from the animal care facility of the Alexandria Faculty of Medicine, Alexandria, Egypt, and were left for acclimatization and observation for one week before the beginning of the study. Rats were housed in standard laboratory conditions with constant temperature, humidity, and 12-h on/off light cycles, and had free access to standard rodent chow and water. Rats were randomly divided into four groups (n = 8 in each group): normal control group, nicotine plus vitamin D3 (VDN) + vehicle group, VDN + canagliflozin 10 mg/kg (VDN + cana [L]) group, VDN + canagliflozin 20 mg/kg (VDN + cana [H]) group.

### Induction of vascular calcification

VC was established as previously described by Niederhoffer et al., (Niederhoffer et al. 1997) with slight modification. On the first day of the study, rats were given intragastric nicotine (0.5 mg/kg; Sigma Chemical Co., St. Louis, MO, USA, 54–11-5) dissolved in 1 ml peanut oil twice daily at 9:00 a.m. and 5:00 p.m., and a single intramuscular injection of vitamin D3 (300,000 IU/kg; Devarol-S ampoules, Memphis Co. for pharm. & Chemical Ind, Egypt) at 9:00 a.m. The normal control group was given an equal volume of peanut oil orally, and an intramuscular injection of saline following the same schedule. From the second day of the study, canagliflozin-treated groups were given 10 mg/kg or 20 mg/kg of canagliflozin (Invokana tablets, Janssen Pharmaceuticals, Inc, Titusville, New Jersey) dissolved in saline orally, daily for four weeks. While, normal and VDN control groups were given the same volume of the drug vehicle orally till the end of the study. The administrated dose of nicotine applied in our study (0.5 mg/kg) was selected based on an initial pilot study that showed that nicotine in higher doses than the selected one (0.75, 1.5, 3, 6, 12.5, 25 mg/kg) were associated with convulsion and death of > 90% rats immediately after administration.

### Measurement of body weight

At the beginning and the end of the study, rats were weighed to calculate the change in body weight over the study period.

### Urine samples collection

Rats were placed in metabolic cages, on the day before scarification, for collection of 24-h urine. Urine volumes were calculated, and samples were stored at − 80 °C until assayed.

### Blood samples collection and harvesting kidney and aorta specimens

On the last day of the experiment, rats were anesthetized, and bloods were drawn from the ophthalmic venous plexus using a fine-walled Pasteur pipette and kept to clot at room temperature. Afterward, serum was obtained by centrifugation at 1000 × g for 15 min and stored at -80 °C. After euthanization, the whole aorta was dissected and divided into three parts; thoracic aorta was fixed in 10% neutral-buffered formalin and abdominal aorta was divided into 2 parts and frozen at -80 °C. Both kidneys were collected; one kidney was stored in 10% neutral-buffered formalin while the second was kept at -80 °C.

### Histopathological examination of aorta and kidney

About 3–4 mm of fixed aorta rings and kidney tissues from 8 rats in each group were embedded in paraffin blocks, cut into 4–6 μm sections, dewaxed with xylene and hydrated by passing a series of graded ethanol concentrations. Then, sections were stained with hematoxylin and eosin (H&E) staining for microscopic examination by Leica microscope (CH9435 Hee56rbrugg; Leica Microsystems, Switzerland). For detection of VSMC calcification, aorta sections were placed with 2.5% silver solution in bright sunlight after deparaffinization and hydration. Afterwards, sections were rinsed in distilled water. Counterstain was applied via Nuclear-fast Red for 5 min. Lastly sections were washed, dehydrated, cleared, and covered to be examined under light Leica microscope (CH9435 Hee56rbrugg; Leica Microsystems, Switzerland). Histopathological scoring for aortic wall thickness was also evaluated via Leica QWin 500 image analyzer computer system (England). Records were statistically described in terms of mean ± SEM.

### Serum and urinary biochemistry

Serum and urinary urea (Bio-diagnostic, Giza, Egypt, UR 21 10) and creatinine (Bio-diagnostic, Giza, Egypt, CR 12 51), and serum albumin (Bio-diagnostic, Giza, Egypt, AB 10 10) were assayed using commercial kits. Serum and urinary calcium and phosphorus levels (Egyptian Co. for Biotechnology – Spectrum Diagnostics, Al-Obour city, Egypt, 226,002, 294,002 for calcium and phosphorus, respectively) were estimated according to the o-cresolphthalein complex-one and UV-phosphomolybdate methods, respectively. Creatinine clearance (CCr) was calculated according to the following equation: CCR (mL/min) = urinary creatinine (UCr; mg/dL) × urine flow (mL/min)/ creatinine in serum (mg/dL). Urine flow per min was estimated by dividing urine volume during 24 h by 1,440.

### Measurement of calcium content in aorta and kidney

Portions of descending aorta and left kidney samples were dried at 55 °C for 2 and 6 h, respectively. Calcium was extracted with 10% formic acid (30 µl/mg of dry tissue), and left overnight at 4 °C. The colorimetric o-cresolphthalein complexone method was used to determine calcium levels in acid tissue extracts and normalized to weights of dry tissues (Egyptian Co. for Biotechnology – Spectrum Diagnostics, Al-Obour city, Egypt, 226,002).

### Aorta homogenization and total protein estimation

Aortic tissue was homogenized in lysis buffer (150 mM NaCl, 10 mM Tris solution, 1% Triton X-100, pH 7.4) with protease inhibitor (Sigma-Aldrich, St. Louis, MO, USA, 11,836,170,001) and centrifuged (10,000 × g, 10 min at 4 °C). The supernatant was kept at -80 °C for further analysis. Total protein content was determined according to Lowry's method (LOWRY et al. 1951).

#### Measurement of ALP activity

The activity of ALP in aortic tissue was measured using the ALP assay kit as instructed (Bio-diagnostic, Giza, Egypt, AP 10 20). In short, the aortic tissue homogenate was mixed 1:1 with a reaction mixture containing stock substation solution and alkaline buffer. After adding 1.5 ml of developer solution, the mixture was incubated at 37 °C for 15 min. At 520 nm, the absorbance of each well was measured using a microplate reader. The established standard curve was used to estimate the ALP levels in the specimens.

#### Lipid peroxidation assay

The lipid peroxidation process in aortic homogenate was assessed using commercial kit (Bio-diagnostic, Giza, Egypt, MD 25 29). The aorta homogenate was heated at 100 °C for 60 min with 1.5 ml of acetic acid (15%), 1.5 ml of thiobarbituric acid (0.8%), and 0.2 ml of sodium dodecyl sulphate (8.1%). After the mixture had cooled, 5 mL of n-butanol-pyridine (15:1) and 1 mL of distilled water were added and vortexed. The organic layer was separated after 10 min of centrifugation at 1200 g, and absorbance was measured at 532 nm with an ELISA plate reader. Malondialdehyde (MDA) is a lipid peroxidation end product that forms a pink chromogen–thiobarbituric acid reactive molecule when combined with thiobarbituric acid (Ohkawa et al. 1979).

#### Assessment of Glutathione (GSH) level

The GSH level in aortic homogenate was measured using commercial kit (Bio-diagnostic, Giza, Egypt, GR 25 11) as previously described (Wheeler et al. 1990). 100 µl of sample, distilled water, or GSH were mixed with 100 µl of sulphosalicylic acid (4%) and stored at 4 °C for at least 1 h before centrifugation at 1200 g for 10 min at 4 °C. The supernatant was then combined with 2.7 ml phosphate buffer (0.1 M, pH 7.4) and 0.2 ml 5,5'-dithiobis-2-nitrobenzoic acid (DTNB) and incubated for 5 min. At 412 nm, the resulting yellow color was measured. Using standard GSH, a standard curve was created. Finally, the GSH content per mg of protein was calculated.

#### Assessment of Superoxide dismutase level (SOD)

SOD activity was measured using available kit (Bio-diagnostic, Giza, Egypt, SD 25 21) following the previously described method (Wheeler et al. 1990). The assay mixture included 0.1 ml supernatant, 1.2 ml sodium pyrophosphate buffer, pH 8.3; 0.052 M, 0.3 ml nitro blue tetrazolium (300 µm), 0.1 ml phenazine methosulphate (186 µm), and 0.2 ml NADH (750 µm). The reaction was started with NADH. After 90 s of incubation at 30 °C, the reaction was halted by adding 0.1 ml glacial acetic acid. The reaction mixture was then vigorously stirred after adding 4.0 ml of n-butanol. The color intensity of the chromogen in butanol was measured spectrophotometrically at 560 nm, and SOD concentration was reported in U/mg protein.

#### Quantification of RUNX and BMP-2

RUNX (MBS2509507, MyBioSource, San Diego, USA) and BMP-2 (MBS701336, MyBioSource, San Diego, USA) levels were detected in aorta using rat specific enzyme-linked immunosorbent assay (ELISA) kits following the manufacture’s instruction. The detection sensitivity was 0.188 ng/m, and 1.95 pg/ml for RUNX and BMP-2, respectively. In short, standards and samples were pipetted into wells and incubated. The detection antibodies were added and incubated after washing. After adding horseradish peroxidase (HRP) conjugate and following substrate solution incubation, immune activity was viewed. After the reaction was stopped, the values were measured at 450 nm with a microplate reader.

#### Western Blot Analysis

For determination of protein expression, aortic tissues were homogenized on ice in RIPA buffer and centrifuged at 10,000 rpm for 30 min for separation of the supernatant. The bicinchoninic acid (BCA) protein assay was used to determine protein content. Next, 50 µg protein lysates from each sample were mixed with 2 × loading buffer, boiled for 5 min, and cooled at 4ºC. Samples were separated by 12% SDS-PAGE mini-gel and run at 120 V. Proteins were transferred to a nitrocellulose membrane at 22 V overnight at 4ºC. The membrane was washed three times with Tris-buffered saline containing Tween 20 (TBST), soaked in blocking buffer for 1 h at RT, and incubated overnight with primary antibodies for AMPK (4150), p62 (39,786), LC3 (2775), and β-actin (4970) (Cell signaling technology, Beverly, MA, USA) diluted with TBST and 5% bovine serum albumin (BSA). After 3 times wash with TBST, the membrane was incubated for 1 h at RT with secondary antibody. Membranes were washed again three times with TBST, and bands were detected by Alkaline phosphatase solution. Finally, the bands were quantified using ImageJ quantification software (Burnette’ 1981).

### Statistical analysis

Statistical analysis was conducted by Statistical package for social science (SPSS) program version 21.0 (SPSS Inc, Chicago, IL). After testing normality of the data, one-way ANOVA test and Tukey post hoc test were used to compare between parametric data, while Kruskal–Wallis test followed by Dunn’s multiple comparisons for non-parametric data. While Pearson coefficient (r) was used to test correlation between two normally distributed quantitative variables. Data were presented as mean ± standard error (SE) or median and inter quantile range (IQR) (25th [Q1] and 75th [Q3] percentiles) and a significant difference was considered as p < 0.05.

## Results

### Effect of canagliflozin on change of body weight

The weight gain in VDN-group was significantly less than the normal control group (p < 0.05). Canagliflozin treatment resulted in more gain in body weight, although it remained significantly less than that of normal control (p < 0.05, Table 1).

**Table 1:** Effect of canagliflozin on body weight, serum and urinary biochemical parameters

	Control	VDN	VDN + Cana [L]	VDN + Cana [H]	P-value
Weight change (g)	25.00 (22.00,29.25)	6.5 (5.00,16.00)^*^	10.00 (7.75, 15.00)^*^	13.50 (8.75, 15.75)^*^	0.0004
Serum creatinine (mg/dl)	0.64 ± 0.04	0.77 ± 0.06	0.71 ± 0.04	0.65 ± 0.37	0.183
Serum urea (mg/dl)	25.75 ± 0.25	27.75 ± 0.53	27.63 ± 0.56	26.87 ± 0.81	0.075
Serum albumin (mg/dl)	3.24 ± 0.211	2.87 ± 0.15	3.44 ± 0.21	3.03 ± 0.13	0.151
Serum calcium (mg/dl)	8.24 ± 0.15	8.46 ± 0.23	8.41 ± 0.20	8.39 ± 0.14	0.851
Serum phosphorus (mg/dl)	6.00 ± 0.15	6.39 ± 0.18	6.34 ± 0.22	6.31 ± 0.14	0.400
U. calcium (mg/dl)	1.03 ± 0.11	0.60 ± 0.11	1.03 ± 0.16	1.00 ± 0.13	0.072
U. phosphorus (mg/dl)	21.43 ± 1.71	9.42 ± 0.73^*^	14.67 ± 1.03^*#^	19.96 ± 1.37^#≡^	< 0.001
Creatinine clearance (mg/min)	0.17 (0.14,0.57)	0.10 (0.08,0.14)^*^	0.51 (0.33,0.85)^#^	0.71 (0.60,0.86)^#^	< 0.001

Data are represented as the mean ± SEM or median (Q1,Q3) of eight animals in each group. Cana [H]; canagliflozin high dose, Cana [L]; canagliflozin low dose, VDN; vitamin D + nicotine. * *P* < 0.05 compared to control group, # P < 0.05 compared to VDN group, ≡ P < 0.05 compared to VDN + Cana [L] group

### Effect of canagliflozin on serum and urine parameters

Table 1 depicts that serum urea, creatinine, albumin, phosphorus, calcium, and urinary calcium levels were non significantly different among the tested groups (p > 0.05). However, there was a significant decrease of urinary creatinine clearance and excretion of phosphorus in VDN group compared to normal rats (p < 0.05). Administration of canagliflozin improved creatinine clearance and phosphorus excretion as compared to the VDN group (p < 0.05).

### Effect of canagliflozin on aortic reactive oxygen species (ROS) production

**Fig. 1:**
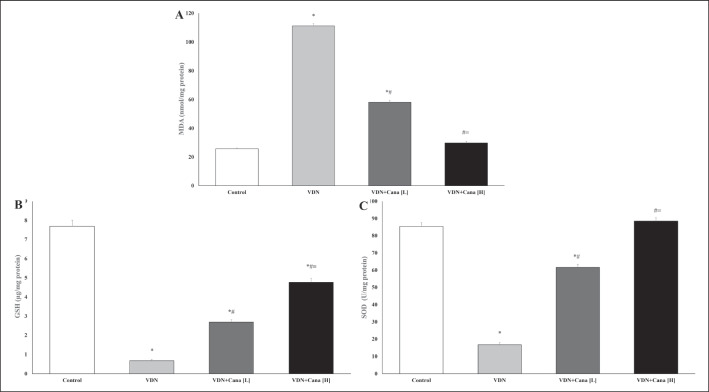
Effects of canagliflozin on markers of oxidative stress in aortic tissues. Treatment of VDN exposed rats with canagliflozin significantly decreased MDA (**A**), increased GSH (**B**) and SOD (**C**) in rat aortas. Cana [H]; canagliflozin high dose, Cana [L]; canagliflozin low dose, GHS; glutathione, MDA; malondialdehyde, SOD; superoxide mutase VDN; vitamin D + nicotine. Data are represented as the mean ± SEM of eight animals in each group. * P < 0.05 compared to control group; # P < 0.05 compared to VDN group; ≡ P < 0.05 compared to VDN + Cana [L] group

Administration of VDN induced aortic oxidative stress as evidenced by a significant increase in MDA and decrease in GSH content and SOD activity in comparison with the normal control group (p < 0.05). Treatment with canagliflozin dose-dependently attenuated the production of MDA and elevated the antioxidants in the aorta relative to the calcification control group (p < 0.05, Fig. 1A-C).

### Effect of canagliflozin on aortic tissues morphology

**Fig. 2:**
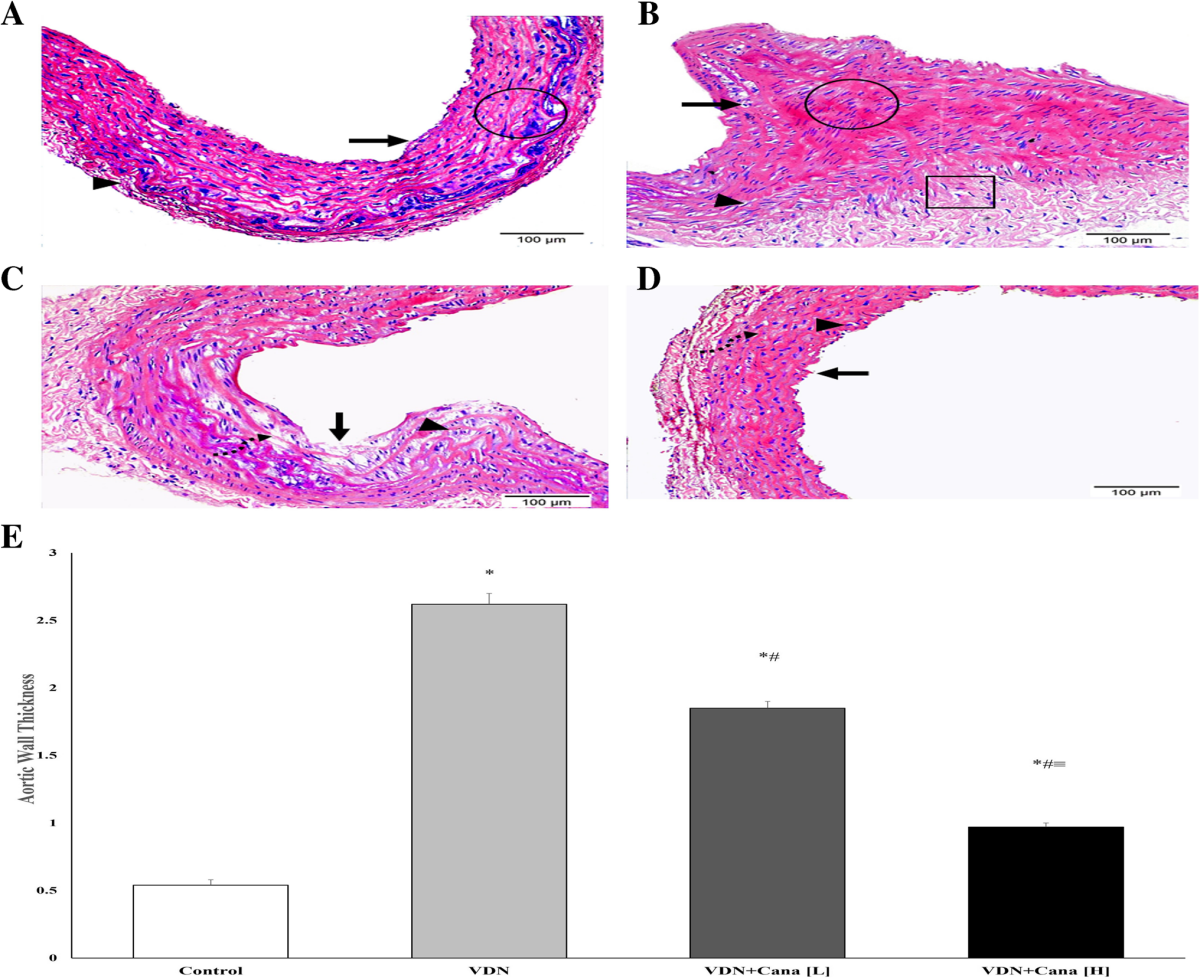
Representative photomicrographs of the histopathological features of the aortic tissues stained with H&E (Mic. Mag × 200). (**A**) Control group displaying the standard histological assembly of aortic layers; tunica intima (arrow), tunica media (circle), and tunica adventitia (arrowhead). (**B**) VDN-group shows an increase in aortic thickness associated with foamy macrophage (arrow), successive aggregation of smooth muscle cells and collagen fibers (circle), fragmented elastic fibers (arrowhead), as well as necrotic core (square). (**C**) VDN + Cana [L] group displaying amelioration of aortic architecture with some areas of degeneration in tunica intima, sloughing of endothelial layer (arrow), dispersion of foamy macrophage and inflammatory cells (arrowhead), as well as limited fragmentation of elastic fibers (dashed arrow). (**D**) VDN + Cana [H] group showing evident improvement of aortic layers; tunica intima (arrow), a few numbers of foamy macrophages and inflammatory cells along the subendothelial layer (arrowhead), and regular elastic fibers (dashed arrow). (**E**) Statistical analysis of aortic wall thickness (micron). Cana [H]; canagliflozin high dose, Cana [L]; canagliflozin low dose, VDN; vitamin D + nicotine. Data are represented as the mean ± SEM of eight animals in each group. * P < 0.05 compared to control group; # P < 0.05 compared to VDN group; ≡ P < 0.05 compared to VDN + Cana [L] group

H&E staining of aortic rings of normal control group showed normal histological features of the aorta. On the other hand, non-treated aortic sections from VDN-calcification group showed distorted morphology of the aorta in the form of aortic thickness associated with foamy macrophages, aggregation of smooth muscle cells, collagen fibers, and fragmented elastic fibers as well as necrotic core. The low dose of canagliflozin partially alleviated the normal aortic architecture, although degeneration of some areas of the tunica intima, sloughing of the endothelial layer, and infiltration with foamy macrophages and inflammatory cells were still observed. On the other hand, the high dose of canagliflozin nearly resorted normal appearance of the tunica intima and elastic fibers (Fig. 2A-D).

### Effect of canagliflozin on kidney morphology

**Fig. 3:**
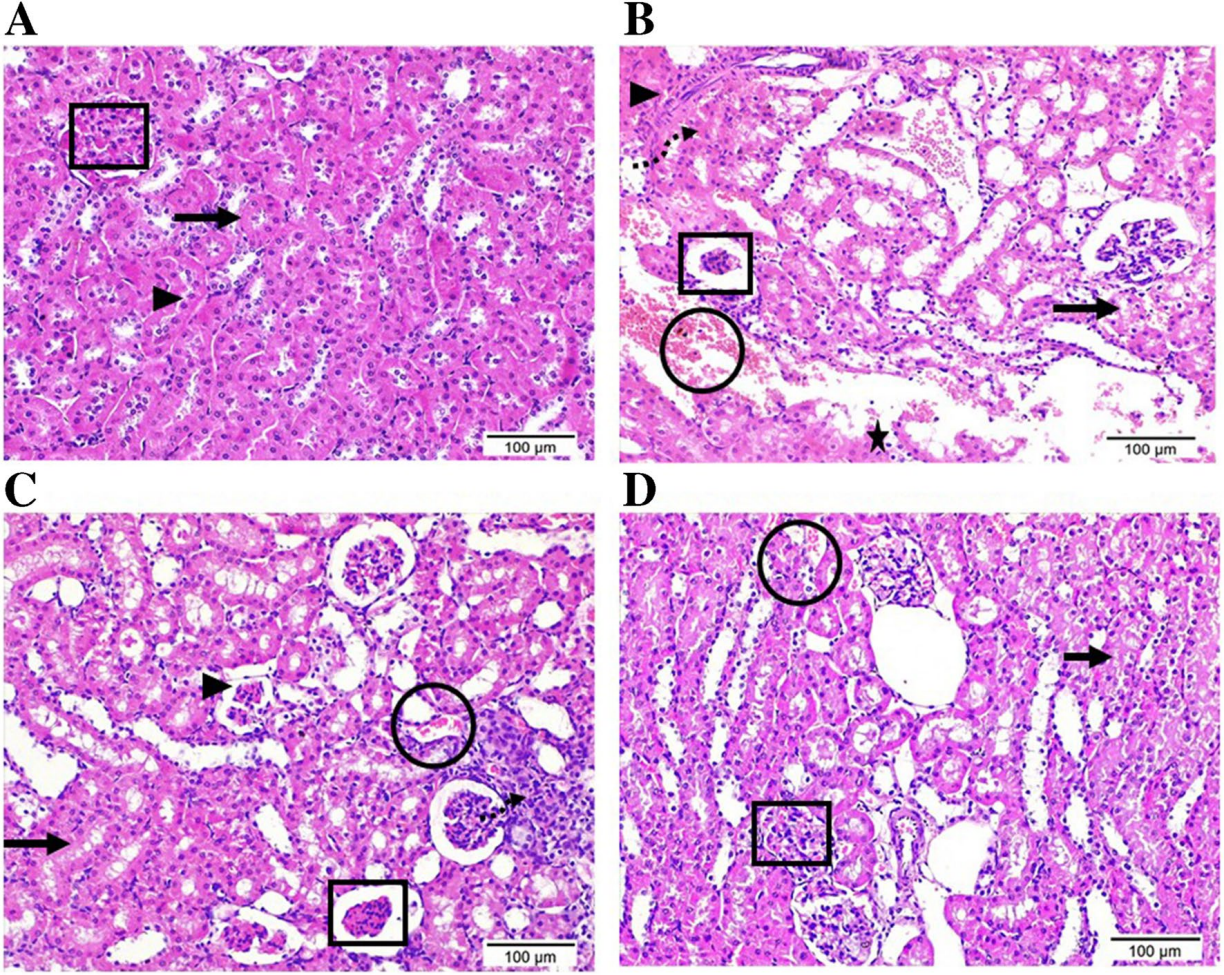
Photomicrographs display the histopathological alterations in kidney tissue sections stained with H&E (Mic. Mag × 200). (**A**) Control group shows the normal histological structure of renal cortex enclosing renal corpuscle with intact bowman’s capsule and glomerulus (square), proximal (arrow) and distal (arrowhead) convoluted tubules. (**B**) VDN group highlights serious renal damage evidenced by atrophy of the renal corpuscle encircled by high inflammatory cells (square), interstitial fibrosis (arrowhead), hemorrhage (circle), necrotic areas (dashed arrow), as well as obvious edema leading to dispersion between renal tubules (triangle). Renal tubules exist with dilatation, hyalinization (star), and others with deterioration, desquamation of the epithelial lining, and cytoplasmic vacuolization with pyknotic nuclei of the lining epithelium (arrow). (**C**) VDN + Cana [L] group showing a considerable tissue recovery, but still some renal corpuscles appear atrophied (square), a few bowmen’s capsule noticed with a very thin thickness (arrowhead), and elevated number of inflammatory cells (dashed arrow) and some collapsed renal tubules with epithelial desquamation (arrow). (**D**) VDN + Cana [H] group revealing obvious improvement with a restoration of most histological structure of renal cortex area. Renal corpuscles are nearly normal in structure (square) while a small area of interstitial hemorrhage as well as a few collapsed renal tubules with epithelial desquamation (arrow) were still noticed

Kidney specimens from the VDN group showed disrupted renal morphology in the form of atrophy of renal corpuscles, interstitial fibrosis and tubular dilatation, hyalinization and desquamation of its epithelial lining as well as cytoplasmic vacuolization with pyknotic nuclei of the lining epithelium. Moreover, it revealed excessive infiltration of inflammatory cells and areas of hemorrhage and necrosis. In line with the observed improvement of aortic tissues, canagliflozin dose-dependently improved the renal damage induced by VDN (Fig. 3A-D).

### Effect of canagliflozin on calcium content of aorta and kidney

**Fig. 4:**
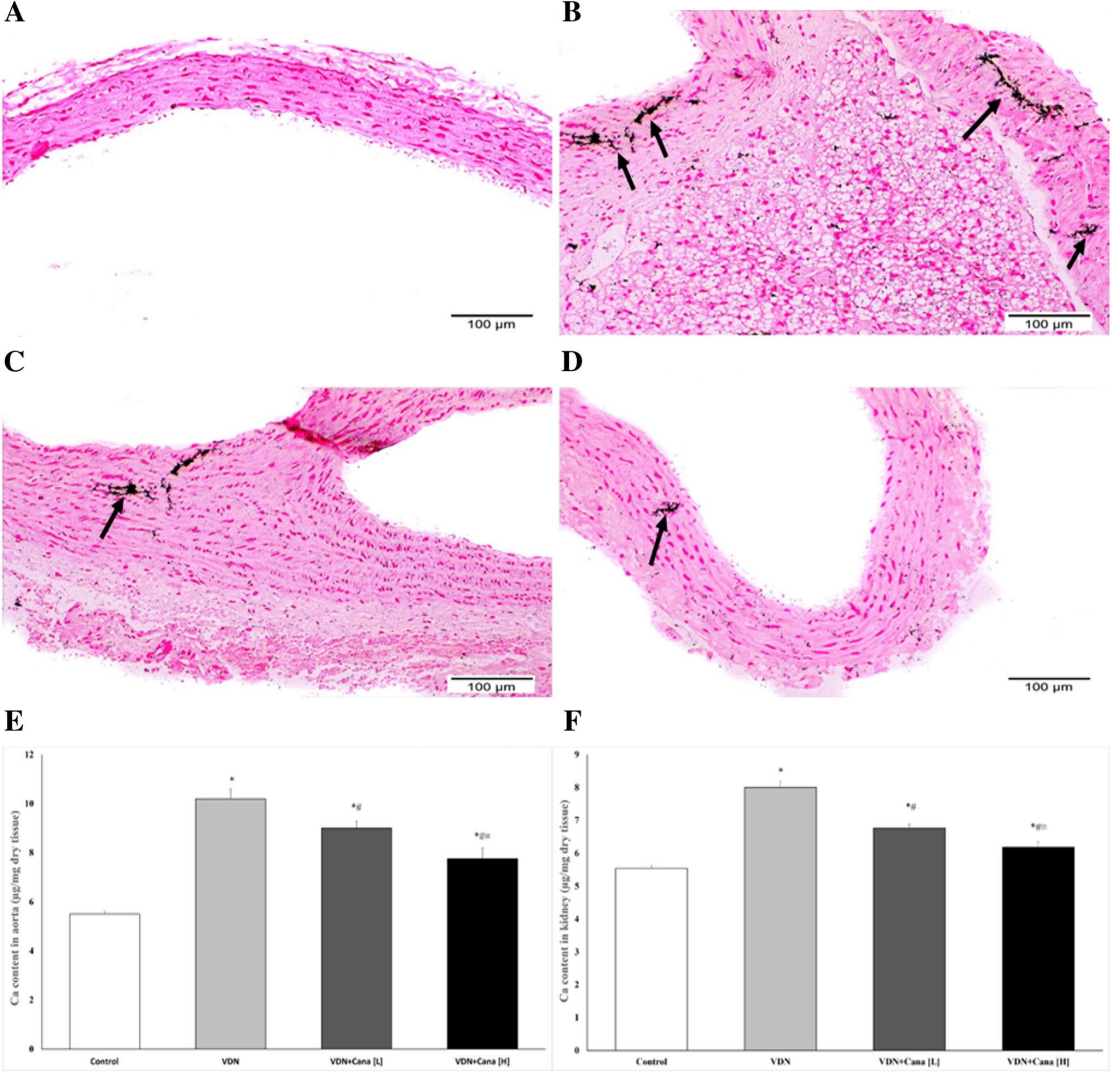
Canagliflozin attenuates tissue calcification in VDN-treated rats. Figures A-D; representative photomicrographs of aortic rings stained by von Kossa stain. (**A**) Control group shows no calcium deposits along aortic wall, (**B**) VDN group shows evident black calcium deposits mainly homing the tunica media, while only a few of them appeared in the tunica intima (arrows). (**C**) VDN + Cana [L] appears with a moderate quantity of calcium deposits along tunica intima as well as media of aortic wall (arrow). (**D**) VDN + Cana [H] demonstrates a few amounts of calcium deposits in tunica media of aortic wall (arrow). Figures E&F display chemical analysis of calcium content of aorta (**E**) and kidney (**F**). Cana [H]; canagliflozin high dose, Cana [L]; canagliflozin low dose, VDN; vitamin D + nicotine. Data are represented as the mean ± SEM of eight animals in each group. * P < 0.05 compared to control group; # P < 0.05 compared to VDN group; ≡ P < 0.05 compared to VDN + Cana [L] group

Histopathological examination of the aortic rings of non-treated VDN exposed rats stained with Von Kossa revealed high calcium deposits primarily in the tunica media, with only a few deposits appearing in the tunica intima. The calcification was markedly reduced by canagliflozin treatment, particularly at the high dose that resulted in few calcium deposits (Fig. 4A-D).

Chemical analysis of calcium levels in aortic and kidney tissues runs in parallel to the histological findings. It revealed a significant increase in calcium content of the aorta and the kidney of the VDN non-treated group when compared to normal control rats (p < 0.05). Rats treated with either low or high doses of canagliflozin exhibited a significant reduction in the calcium content of the aorta and the kidney as compared to the VDN group (p < 0.05). it is worthy to note that the higher dose of canagliflozin resulted in as significant decrease in comparison to the low dose (p < 0.05, Fig. 4E & F).

### Effect of canagliflozin on osteogenic differentiation markers

**Fig. 5:**
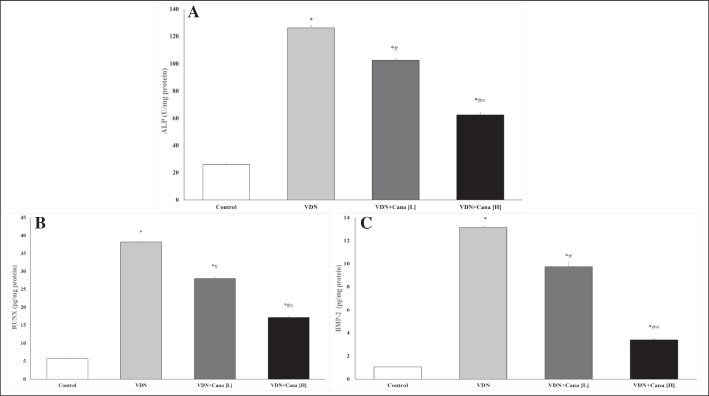
Canagliflozin attenuates VDN-induced osteogenic differentiation. The aortic levels of ALP (**A**), RUNX (**B**) and BMP-2 (**C**) are significantly reduced after treatment with canagliflozin (10 mg/kg or 20 mg/kg) for 4 weeks. Cana [H]; canagliflozin high dose, Cana [L]; canagliflozin low dose, VDN; vitamin D + nicotine. Data are represented as the mean ± SEM of eight animals in each group. * P < 0.05 compared to control group, ^#^ P < 0.05 compared to VDN group, ^≡^ P < 0.05 compared to VDN + Cana [L] group

The vascular calcification group had higher ALP activity, a hallmark of active osteoblast, than the normal control group (Fig. 5, p < 0.05). A comparable increase in RUNX and BMP-2 levels were also noted in aortic tissues of VDN group. Both the low and high doses of canagliflozin decreased ALP activity, RUNX and BMP-2 levels compared to VDN-group (Fig. 5, p < 0.05), meanwhile, they remained significantly higher than the normal control group (p < 0.05).

### Effect of canagliflozin on protein expression of AMPK, LC3B and P62

**Fig. 6:**
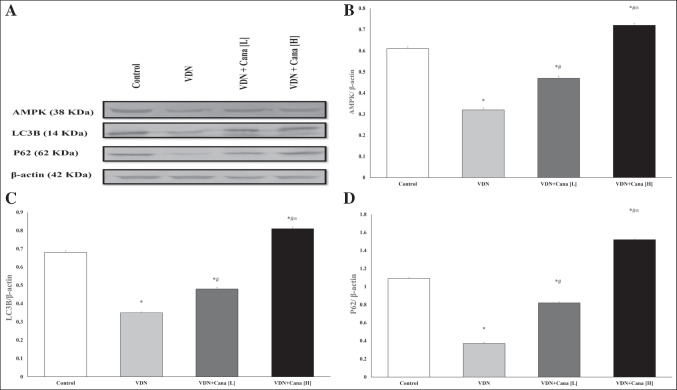
Canagliflozin increased AMPK expression and restores autophagy markers in rat aortas. (**A**) Representative Western blot bands of AMPK, LC3B and P62 in rat aortas. (**B**-**D**); the semiquantitative analysis of protein expression of AMPK, LC3B and P62 adjusted to the expression of β-actin. Cana [H]; canagliflozin high dose, Cana [L]; canagliflozin low dose, VDN; vitamin D + nicotine. Data are represented as the mean ± SEM of eight animals in each group. * P < 0.05 compared to control group, ^#^ P < 0.05 compared to VDN group, ^≡^ P < 0.05 compared to VDN + Cana [L] group

Figure 6 shows representative bands of the expression of AMPK and autophagy markers, LC3B and P62, and their relative intensities. VDN induced a substantial decrease in protein expression of AMPK and autophagic markers compared with normal control (p < 0.05). Both doses of canagliflozin significantly enhanced the expression of AMPK, and autophagic markers as compared with non-treated VC group. Interestingly, the high dose of canagliflozin exhibited more rise in their expression relative to normal rats (p < 0.05).

### Correlation between studied parameters

**Fig. 7:**
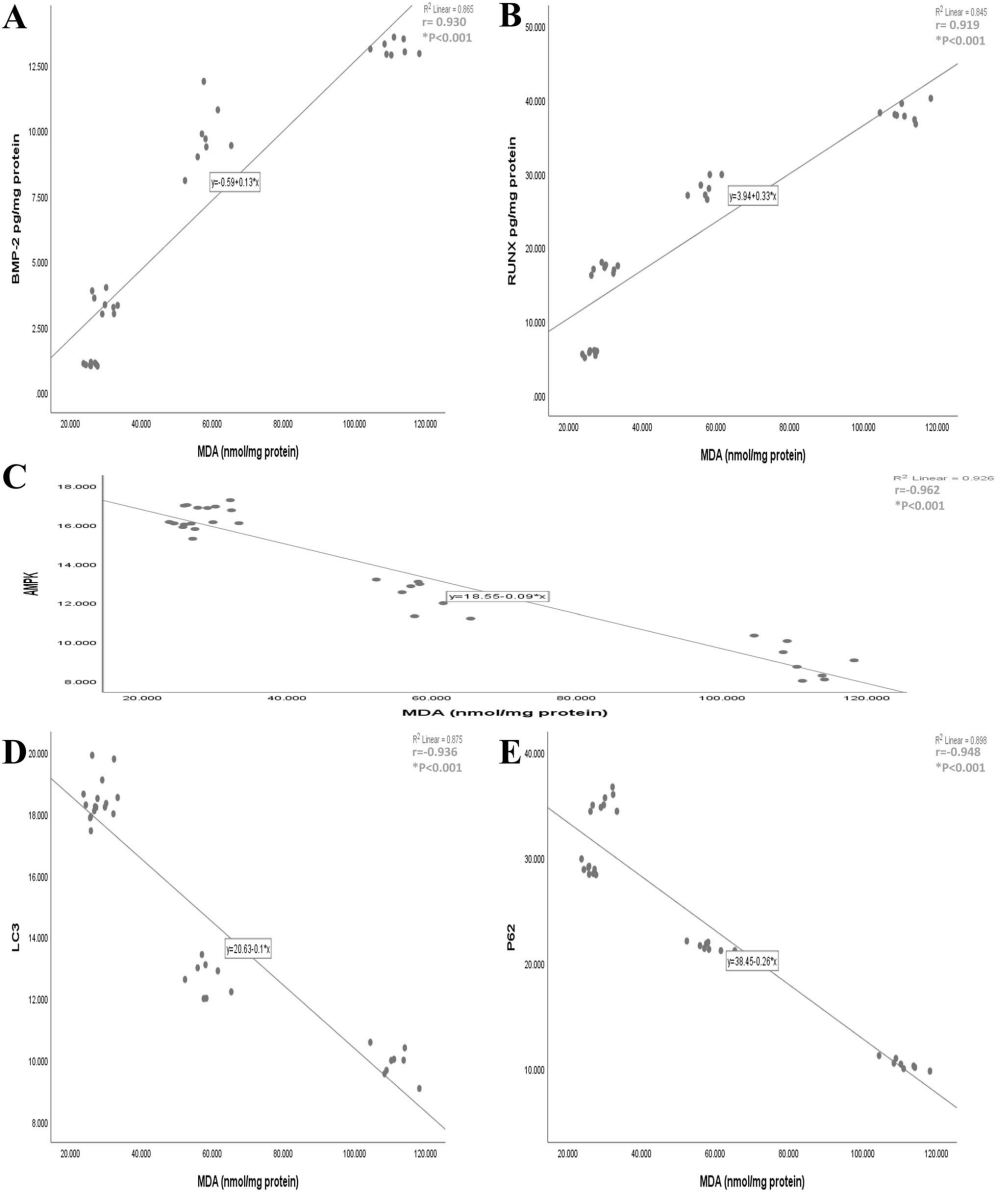
Correlation between studied parameters. **A** and **B**; positive correlation between BMP-2, RUNX, and MDA. **C**; negative correlation between AMPK and MDA. **D** and **E**; negative correlation between autophagic markers and MDA in rat aortas

Results showed that MDA was positively correlated with osteogenic markers BMP-2 and RUNX (r = 0.930, and 0.919, respectively, p < 0.001). AMPK, LC3, and p62 showed significant negative correlation with MDA (r = -0.962, -0.936, -0.948, respectively, p < 0.001). This finding indicates that increased activity of AMPK and autophagy would be associated with attenuation of oxidative stress and further inhibition of aortic calcification (Fig. 7).

## Discussion

Vascular calcification (VC) is a major predisposing factor for cardiovascular events owing to its deleterious effects on vascular function. It is not a merely passive process of calcium deposition in the vessel wall, but it is recognized as an active and well-regulated process that shares some aspects with bone remodeling (Lee et al. 2020). The present study employed the VDN-induced rat model to investigate the possible protective effect of canagliflozin on VC. VDN model is known to produce arterial calcification that shows common pathological features with VC associated with aging, diabetes, and chronic renal diseases (Niederhoffer et al. 1997). We revealed an elevated levels of Runx2 and BMP in calcified vessels. Both of which reflect transition of VSMC into osteogenic phenotype (Lin et al. 2015). BMPs, members of transforming growth factor-beta (TGF-β) superfamily, have been suggested to enhance the expression of Runx2, modulate VSMCs apoptosis, and promote expression of ALP in cultured VSMCs. However, the exact mechanisms of BMPs in promoting VC are still lacking (Cai et al. 2012).

Mechanistically, hypervitaminosis D had been shown to upregulate vitamin D receptors that facilitates calcium uptake and deposition in VSMC. In turn, excessive intracellular calcium induces organellar dysfunction and cellular death (Rajasree et al. 2002). The role of nicotine could be explained via multiple mechanisms including induction of oxidative stress and NFκB signaling, deregulation of VSMCs apoptosis, and direct stimulation of VSMCs differentiation via activating the classical nicotine receptor. Collectively, these factors amplify calcium deposition in vascular tissues (Cucina et al. 2008; Wang et al. 2019; Yoshiyama et al. 2014).

Consistent with previous studies (Amer et al. 2021) (Pei et al. 2021), the present results revealed an elevation of the calcium content in the aorta as evidenced by biochemical and histological findings, whereas the observed elevation of ALP activity is an early pathognomonic marker of osteogenic differentiation (Chang et al. 2008). Apart from aorta, the abnormal calcium deposition in the kidneys of VDN control group is known to impose harmful structural and functional changes of kidney tissues (Shi et al. 2018) (Amer et al. 2021). Herein, we detected a significant decrease of urinary creatinine clearance with no significant changes in serum urea, creatinine, phosphorus, and calcium levels. These results were expected as VDN is thought to reflect an early stage of kidney damage without affecting serum electrolytes (Shi et al. 2018).

The present study revealed that canagliflozin decreased aortic and renal calcium content, ALP activity in aorta, and protein expression of RUNX and BMP-2 in a dose-dependent manner. In a linked fashion, canagliflozin ameliorates abnormal histological findings in the aorta. It has been previously demonstrated that SGLT inhibitors show anti-calcific effect in diabetic condition thanks to its hypoglycemic effect, blood pressure lowering effect, renal function ameliorative effect, and anti-inflammatory effect (Ghosh et al. 2020). However, the role of different members of SGLT inhibitors in non-diabetic conditions needs further investigations.

Oxidative stress (OS) plays a crucial role in the induction and progression of VC as evidenced in both in vitro and in vivo studies. Chang et al. (Chang et al. 2008), demonstrated that OS correlates with the expression of Runx2. Furthermore, OS induces the expression of proinflammatory mediators that further promotes the switch of VSMCs into osteogenic phenotype (Shen et al. 2021). Thus, cutting off oxidants production and proinflammatory cytokines could mitigate this vicious circle and ameliorate VC. This notion was further supported by the ameliorative effect of various products with antioxidant properties to ameliorate VC (Mody et al. 2001) (Cui et al. 2017).

The observed beneficial effect of canagliflozin was associated with a significant reduction of MDA, and enhanced expression of SOD and GSH in the calcified aortas of both canagliflozin-treated groups. This result is in a good agreement with previous studies demonstrated a remarkable antioxidant and anti-inflammatory properties of canagliflozin in diabetic and non-diabetic status (Hasan et al. 2020) (Schiattarella and Bode 2021). Sayour et al., showed that a single intravenous bolus of canagliflozin (3 µg/kg bodyweight) attenuated the expression of nitro-oxidative stress genes in the myocardium of non-diabetic rats (Sayour et al. 2019). Kondo et al., demonstrated that the antioxidant effect of canagliflozin in human cardiomyocytes depends on its direct effect on SGLT1 (Kondo Hidekazu et al. 2021). As a support, another study revealed that empagliflozin, a more selective SGLT2 inhibitor, did not affect myocardial redox signaling (Anker and Butler 2018). Those results shed light on the crucial role of SGLT1/SGLT2 affinity in mediating the cardioprotective effects of different members of SGLT2 inhibitors.

The critical role of autophagy in maintaining normal vascular function has recently been explored (Neutel et al. 2020). Several studies demonstrated that targeting VSMCs autophagy could be a novel promising strategy to mitigate VC. Gao et al., found that aldosterone enhanced VC in both in vivo and in vitro model of high phosphate-induced vascular calcification via inhibiting autophagy, whereas rapamycin, an autophagy inducer, could reverse this effect (Gao et al. 2020). Peng et al., revealed that the protective effects of estrogen against VC in VDN model is accomplished by promoting autophagy (Peng et al. 2017).

Hence, we investigated the potential role of canagliflozin in modulating autophagy in calcified tissues to further explore its protective mechanisms in this model. The present study demonstrated, for the first time, that canagliflozin dose dependently upregulated LC3 (a marker of autophagosomes formation) and p62 expression paralleled to decreased Runx2 and BMP in calcified vessels.

The effect of SGLT2 inhibitors on autophagy was initially reported in diabetic animals (Aragón-Herrera et al. 2019; Li et al. 2021). They have been assumed to indirectly activate autophagic flux via induction of fasting like state to regulate various transcription signaling pathways that regulate autophagy (Fukushima et al. 2021).

Recently, series publications reported a link between the protective effects of canagliflozin in different tissues and activation of autophagy. Canagliflozin exerts an anti-inflammatory action via promoting autophagy in both in vivo and in vitro cells (Niu et al. 2022; Xu et al. 2018). Park et al., showed that canagliflozin protects kidneys from cisplatin-induced injury in non-diabetic mice and in HK-2 cells via regulating autophagy (Park et al. 2022). They attributed such protective effect to the activation of AMPK and inactivation of mammalian target of rapamycin (mTOR).

Several studies show that AMP-activated kinase (AMPK) plays a critical role in VC via its direct inhibitory effect on the expression of Runx2 in VSMCs (Cao et al. 2013). Further mechanisms include the inhibition of endoplasmic reticulum stress, regulation of eNOS-NO signaling pathway, and regulation of mitochondrial dynamics (Lu et al. 2021a). Additionally, mounting data suggest AMPK as an upstream regulator of autophagy via direct phosphorylation of ULK1, inactivation of mTORC1, and modulation of autophagosomes formation (Neutel et al. 2020), (Hardie 2011; Kim et al. 2011).

As mentioned above, activation of autophagy can ameliorate the pathological abnormalities in calcified vessels via restoration of cytokines and ROS levels towards normal and inhibition of apoptosis and osteoblastic transformation of VSMCs (Grootaert et al. 2015) (Chen et al. 2020) (Lu et al. 2021b). Metformin, an AMPK activator, downregulated the expression of Runx2 in VSMCs, and this protective effect was blocked by compound C, the pharmacologic inhibitor of AMPK (Cao et al. 2013). Likewise, ghrelin and melatonin activated autophagy and ameliorated VC in an AMPK-dependent manner (Chen et al. 2020) (Xu et al. 2017).

Among various SGLT inhibitors, canagliflozin has a unique feature of directly activating AMPK in concentrations corresponding to that of therapeutic doses (Hawley et al. 2016). The modulatory effect of canagliflozin on AMPK signaling has been shown in adipose tissues (Yang et al. 2020), kidneys, (Park et al. 2022), and human endothelial cells (Mancini et al. 2018). Interestingly, activation of AMPK was not observed with dapagliflozin, empagliflozin, or their natural product phlorizin (Hawley et al. 2016). In a linked fashion, our data from immunoblot analysis showed that canagliflozin induces AMPK in a concentration-dependent manner.

The off-target effects of SGLT2 inhibitors don’t seem to be equal. For instance, Behnammanesh et al., proved that canagliflozin, but not empagliflozin or dapagliflozin, inhibits VSMCs proliferation and migration in pharmacologically relevant concentrations (Behnammanesh et al. 2020). Similarly, Kondo et al., showed that canagliflozin, but not empagliflozin, can suppress myocardial oxidative stress in diabetic and non-diabetic patients (Kondo Hidekazu et al. 2021).

In conclusion, the present study demonstrates a remarkable vascular protective effect of canagliflozin in VDN-calcification. This effect may be related, at least in part, to its ameliorative effect on oxidant production, AMPK activation, and regulation of autophagy. In the present study, we didn’t verify whether other SGLT inhibitors exert similar effects in calcification. So, future studies are warranted to distinguish whether the observed anti-calcific effect is drug-effect or class-effect to optimize the selection of specific SGLT2 inhibitors in various pathological conditions.

### Supplementary Information

Below is the link to the electronic supplementary material.

Supplementary file1 (DOCX 6968 KB)

## Data Availability

All data generated or analyzed during this study are included in this published article.

## Abbreviations

ALP: Alkaline Phosphatase
AMPK: Adenosine monophosphate activated protein kinase;
BMP-2: Bone Morphogenic Protein-2
Cana: Canagliflozin
CCr: Creatinine Clearance
DM: Diabetes mellitus
DTNB: 5,5'-Dithiobis-2-Nitrobenzoic Acid
GSH: Glutathione
MDA: Malondialdehyde
NO: Nitric Oxide
OS: Oxidative Stress
PPi: Inorganic Pyrophosphate
ROS: Reactive Oxygen Species
Runx2: Runt-Related Transcription Factor
SE: Standard Error
SGLT2: Sodium-Glucose Cotransporter-2
SIRT1: Sirtuin-1
SOD: Superoxide Dismutase
TGF-β: Transforming Growth Factor-Beta
UCr: Urinary Creatinine
VC: Vascular Calcification
VSMCs: Vascular Smooth Muscle Cells
VDN: Vitamin D3 Plus Nicotine
