# Supplementary material for: AMPK-mediated autophagy is involved in the protective effect of canagliflozin in the vitamin D3 plus nicotine calcification model in rats
Source: Naunyn Schmiedebergs Arch Pharmacol. 2023 Jul 31;397(2):873–88. doi: 10.1007/s00210-023-02627-x (PMC10791829; doi:10.1007/s00210-023-02627-x)
Supplement: Supplementary file 1 — Supplementary file1 (DOCX 6968 KB) [file 210_2023_2627_MOESM1_ESM.docx]

**AMPK-mediated autophagy is involved in the protective effect of canagliflozin in the vitamin D3 plus nicotine calcification model in rats**

Supplementary Fig 1.: Representative photomicrographs of the histopathological features of the aortic tissues stained with H&E (Mic. Mag ×50).

| **Control group** |  |
| --- | --- |
| **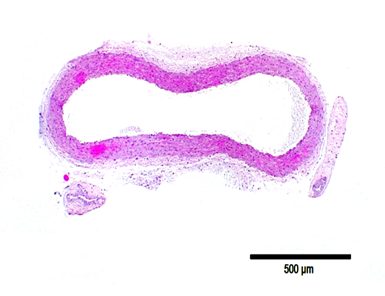** | **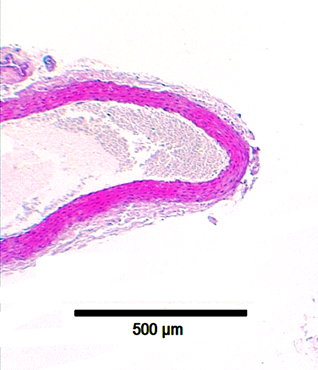** |
| **VDN-group** |  |
| **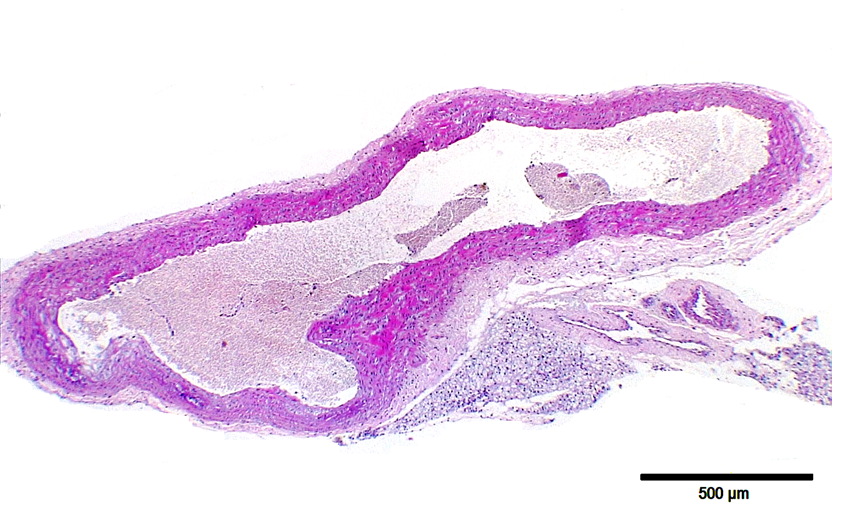** | **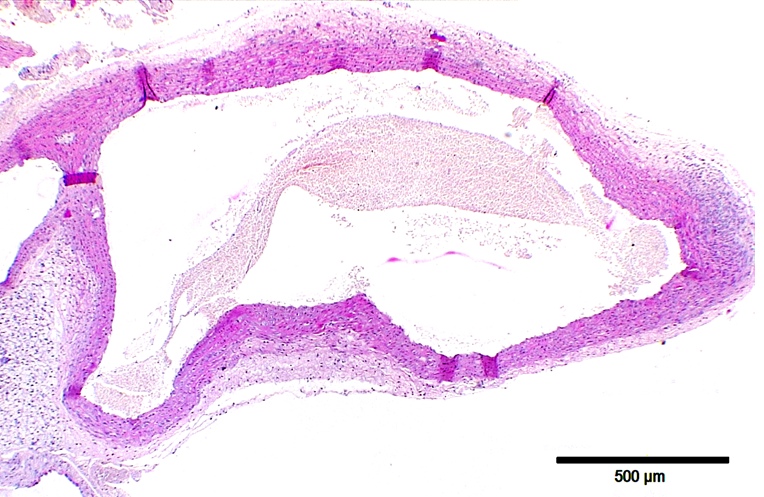** |
|  |  |
| **VDN + Cana [L] group** |  |
| **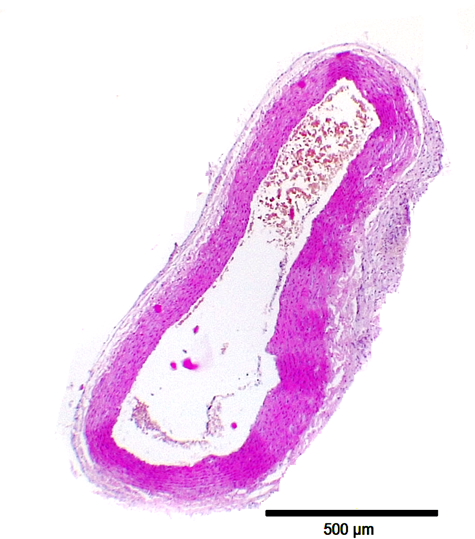** | **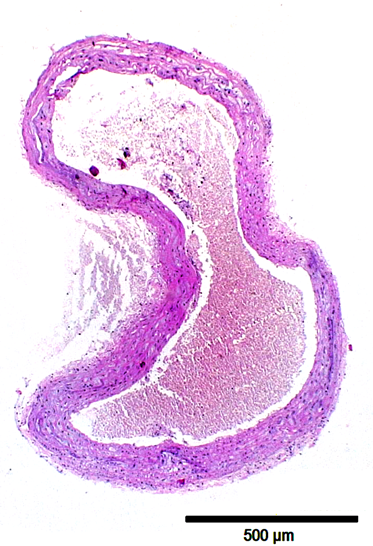** |
| **VDN + Cana [H] group** |  |
| **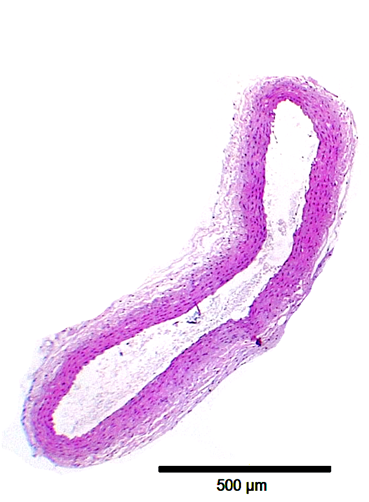** | **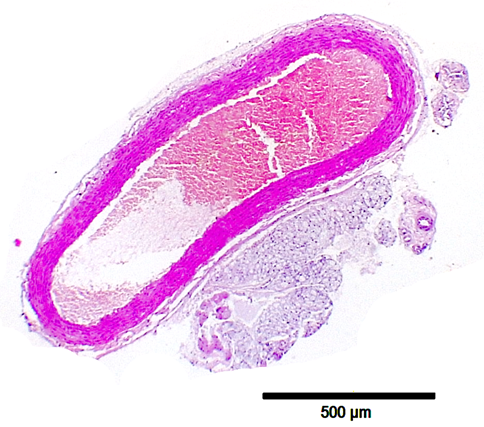** |
